# Robotic-arm assisted total knee arthroplasty is associated with improved accuracy and patient reported outcomes: a systematic review and meta-analysis

**DOI:** 10.1007/s00167-021-06464-4

**Published:** 2021-02-06

**Authors:** Junren Zhang, Wofhatwa Solomon Ndou, Nathan Ng, Paul Gaston, Philip M. Simpson, Gavin J. Macpherson, James T. Patton, Nicholas D. Clement

**Affiliations:** Department of Orthopedics and Trauma, The Royal Infirmary of Edinburgh, Little France, Edinburgh, EH16 4SA UK

**Keywords:** Knee, Arthroplasty, Robotic, Knee alignment, Knee balancing techniques, Outcome, Complications

## Abstract

This systematic review and meta-analysis were conducted to compare the accuracy of component positioning, alignment and balancing techniques employed, patient-reported outcomes, and complications of robotic-arm assisted total knee arthroplasty (RATKA) with manual TKA (mTKA) and the associated learning curve. Searches of PubMed, Medline and Google Scholar were performed in October 2020 using PRISMA guidelines. Search terms included “robotic”, “knee” and “arthroplasty”. The criteria for inclusion were published clinical research articles reporting the learning curve for RATKA and those comparing the component position accuracy, alignment and balancing techniques, functional outcomes, or complications with mTKA. There were 198 articles identified, following full text screening, 16 studies satisfied the inclusion criteria and reported the learning curve of rTKA (*n*=5), component positioning accuracy (*n*=6), alignment and balancing techniques (*n*=7), functional outcomes (*n*=7), or complications (*n*=5). Two studies reported the learning curve using CUSUM analysis to establish an inflexion point for proficiency which ranged from 7 to 11 cases and there was no learning curve for component positioning accuracy. The meta-analysis showed a significantly lower difference between planned component position and implanted component position, and the spread was narrower for RATKA compared with the mTKA group (Femur coronal: mean 1.31, 95% confidence interval (CI) 1.08–1.55, *p*<0.00001; Tibia coronal: mean 1.56, 95% CI 1.32–1.81, *p*<0.00001). Three studies reported using different alignment and balancing techniques between mTKA and RATKA, two studies used the same for both group and two studies did not state the methods used in their RATKA groups. RATKA resulted in better Knee Society Score compared to mTKA in the short-to-mid-term follow up (95%CI [− 1.23,  − 0.51], *p*=0.004). There was no difference in arthrofibrosis, superficial and deep infection, wound dehiscence, or overall complication rates. RATKA demonstrated improved accuracy of component positioning and patient-reported outcomes. The learning curve of RATKA for operating time was between 7 and 11 cases. Future well-powered studies on RATKAs should report on the knee alignment and balancing techniques utilised to enable better comparisons on which techniques maximise patient outcomes.

*Level of evidence* III.

## Introduction

Robotic total knee arthroplasty (TKA) is associated with improved accuracy of prosthesis implantation, and may improve outcomes and implant survival [1, 4, 15, 16]. The MAKO robotic-arm assisted TKA (RATKA) system (Stryker, Kalamazoo, Michigan, USA), in contrast to other robotic systems, is a “semi-active” system that allows the surgeon to interact with the robot during bone preparation, implant alignment and balancing of the knee [11]. These are important surgeon-controlled variables that affect patient outcomes, implant stability and long-term survivorship [5, 45, 53].

Recent systematic reviews comparing robotic TKA with manual TKA (mTKA) have not reported the techniques used to align and balance the knee, which may influence the associated functional outcome and survival of the prosthesis [9]. Furthermore, the only systematic review by Batailler et al. to analyse RATKA in isolation, excluding active systems, did not undertake meta-analysis for the outcomes assessed [2]. Reviews by Batailler et al. and Kayani et al. critically assessed outcomes, but pooled analyses was not conducted and limits interpretation of their data [2, 17]. Cadaveric cases were also included in these reviews, which may not reflect clinical results. Four recent meta-analysis have analysed the effects of robotic TKA on accuracy and functional outcomes when compared to mTKA, but most of the studies included were “fully active” systems [1, 7, 39, 43]. Only seven (31%) of 22 studies reported by Agarwal et al., one (10%) of ten reported by Chin et al., seven of 18 (39%) reported by Onggo et al. and none of the of seven studies reported by Ren et al. were “semi-active” robotic-arm assisted TKA (RATKA) [1, 7, 39, 43]. Therefore, the advantages of RATKA compared to mTKA is not clear due to the heterogeneity of systems included in these studies. Mancino et al. compared the rate of complications, but again also included mostly active systems in their review, which have been associated with a greater rate of complications compared to semi-active systems [29, 40].

The authors hypothesized that RATKA improves accuracy and patient-reported outcomes and has a lower complication rate compared to mTKA. Therefore, this systematic review and meta-analysis was conducted to compare the accuracy of component positioning, alignment and balancing techniques employed, patient-reported outcomes and complications of RATKA with mTKA and the associated learning curve.

## Methods

A search of Medline, PubMed and Google Scholar was performed in October 2020 in line with the Preferred Reporting Items for Systematic Review and Meta-Analysis (PRISMA) statement. The study was registered on the PROSPERO International prospective register of systematic reviews (ID no. CRD42020218706).

All identified article titles and abstracts were screened independently by three authors (JZ, WSN and NDC), with those meeting the inclusion criteria screened further by full-text review. On occasions when it was not clear from the abstract if studies were of relevance, the full text of the article was reviewed. Unanimous consensus was met on the inclusion of proposed studies for full text review amongst the authors (JZ, WSN and NDC). Full text studies were further evaluated against the inclusion and exclusion criteria. The reference lists of included studies were reviewed to ensure no other relevant studies were overlooked.

### Search terms and criteria for inclusion

Search terms included (‘robot’ [All fields] OR ‘robotic’ [All fields] OR ‘robotic surgical procedure’ [MeSH terms] with all entry terms and ‘robotic arm assisted’[All fields]) and (‘total knee’ [MeSH terms], OR ‘arthroplasty, replacement, total knee’ [MeSH terms] OR ‘arthroplasty’[MeSH terms]). A search limit for articles published from 2000 to 2020 was applied. A single search of PubMed and Medline yielded 52 abstracts. Two searches of Google Scholar using the search terms (a) all in title: robot total knee and (b) all in title: robotic total knee yielded 146 articles in total. The criteria for inclusion were published clinical research articles studying robotic total knee arthroplasty and reporting on functional outcomes or patient satisfaction or accuracy of component positioning or learning curve or complications. Studies were excluded if they were case reports, review articles, conference abstracts, non-clinical studies or were not available in the English language. For the purposes of this review, there was a focus on “semi-active” robotic systems and, therefore, “fully active” robotic systems were excluded from analysis.

### Data extraction

The included studies were evaluated for the authors, year of publication, title, where it was published, study design (prospective or retrospective), age of patients, number of patients, follow-up (if applicable), the type of implant used and depending on the aims for the study: patient satisfaction, functional outcome, component accuracy, alignment and balancing techniques, complications and learning curve. In addition, the main conclusion from each study was also recorded. If two studies reported on the same cohort of patients, only the latter more complete cohort would be included in the current analysis.

### Outcome measures

The primary objectives were to report the learning curve, accuracy of component positioning, alignment and balancing techniques, functional outcomes and complications within the included studies. Secondary objectives included presenting the demographic data and implants used across the included articles.

### Quality assessment

Using the NIH Quality Assessment Tool for Observational Cohort and Cross-Sectional Studies, all included publications were reviewed independently for potential risk of bias by three authors (JZ, SN, NDC). The assessment tool uses 14 questions to enable allocation of a score to each article (poor, fair or good). If there was disagreement regarding the scoring of a study, consensus was met after discussion amongst both assessors.

### Statistical analysis

Simple descriptive analysis was performed for (a) learning curve of robotic-arm assisted total knee arthroplasty and for studies comparing (b) the accuracy of component positioning, (c) alignment and balancing techniques, (d) patient reported functional outcomes and (e) complications between robotic-arm assisted and manual total knee arthroplasty. Data were extracted from studies comparing (a) the accuracy of component positioning, (b) patient-reported functional outcomes (Knee Society Scores (KSS), Western Ontario and McMaster University Osteoarthritis Index (WOMAC)) and (c) complications (Manipulation under anaesthesia (MUA), superficial and deep infections, and wound dehiscence) between RATKA and mTKA to enable meta-analysis to be undertaken for these outcomes. The manipulation under anaesthesia (MUA), superficial and deep infections, pin site fractures and wound dehiscence were statistically assessed using Peto and the odds ratio were presented as the effect measure. The accuracy of component positioning, KSS, and the WOMAC scores were assessed using inverse variance and the mean difference was presented as the effect measure. For each outcome variable, 95% confidence intervals were presented. Heterogeneity among the studies were assessed using the *χ*2 test and *I*^2^. A fixed effect model was applied when *I*^2^ < 50%, and a random effects model when *I*^2^ > 50%. A *p* value < 0.05 was considered statistically significant in cases in which trials have no event in one arm or another. The meta-analysis was conducted using Review Manager 5.2 (Cochrane Collaboration, Oxford, UK).

## Results

**Fig. 1:**
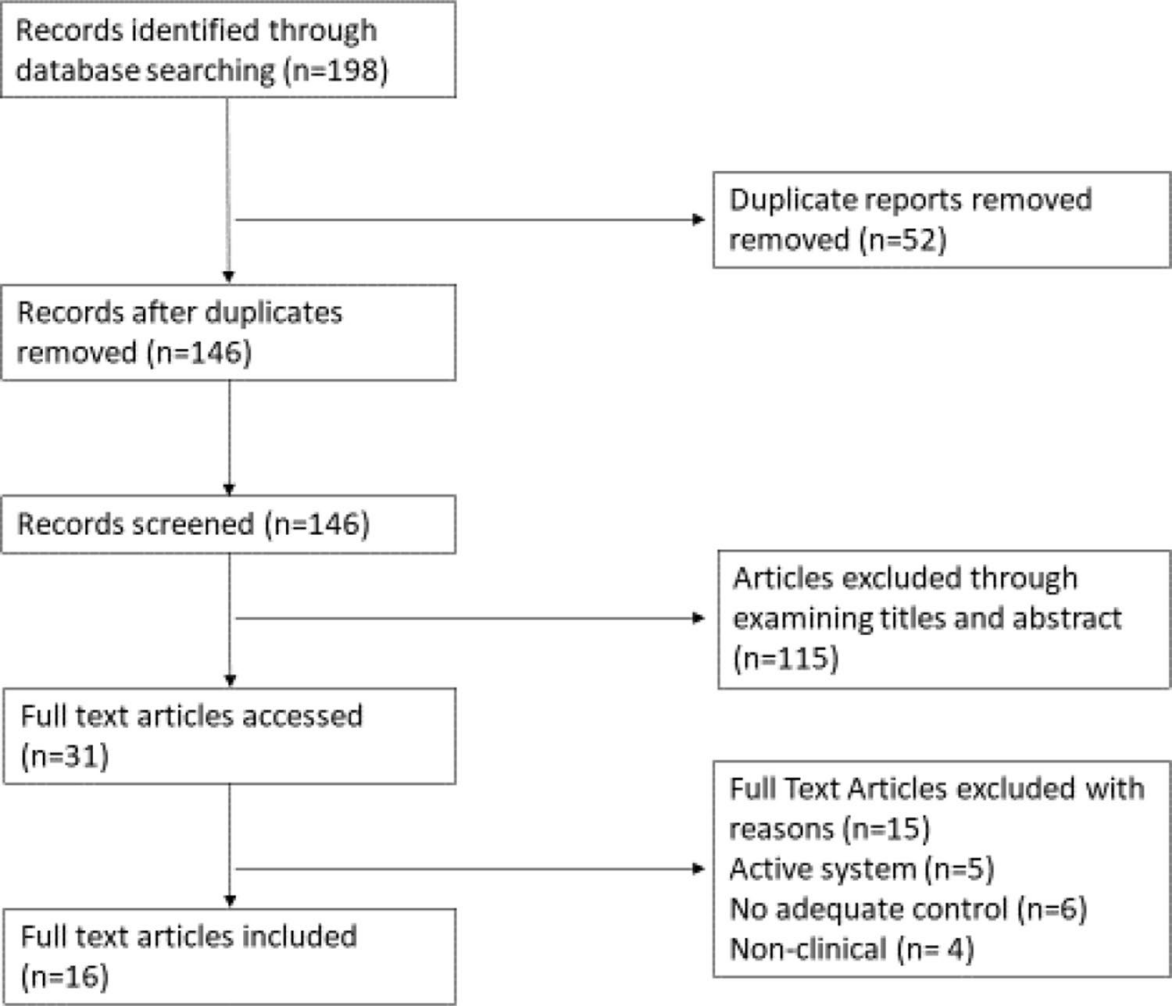
Complete PRISMA flow diagram showing the identification, screening, eligibility and inclusion process

**Fig. 2:**
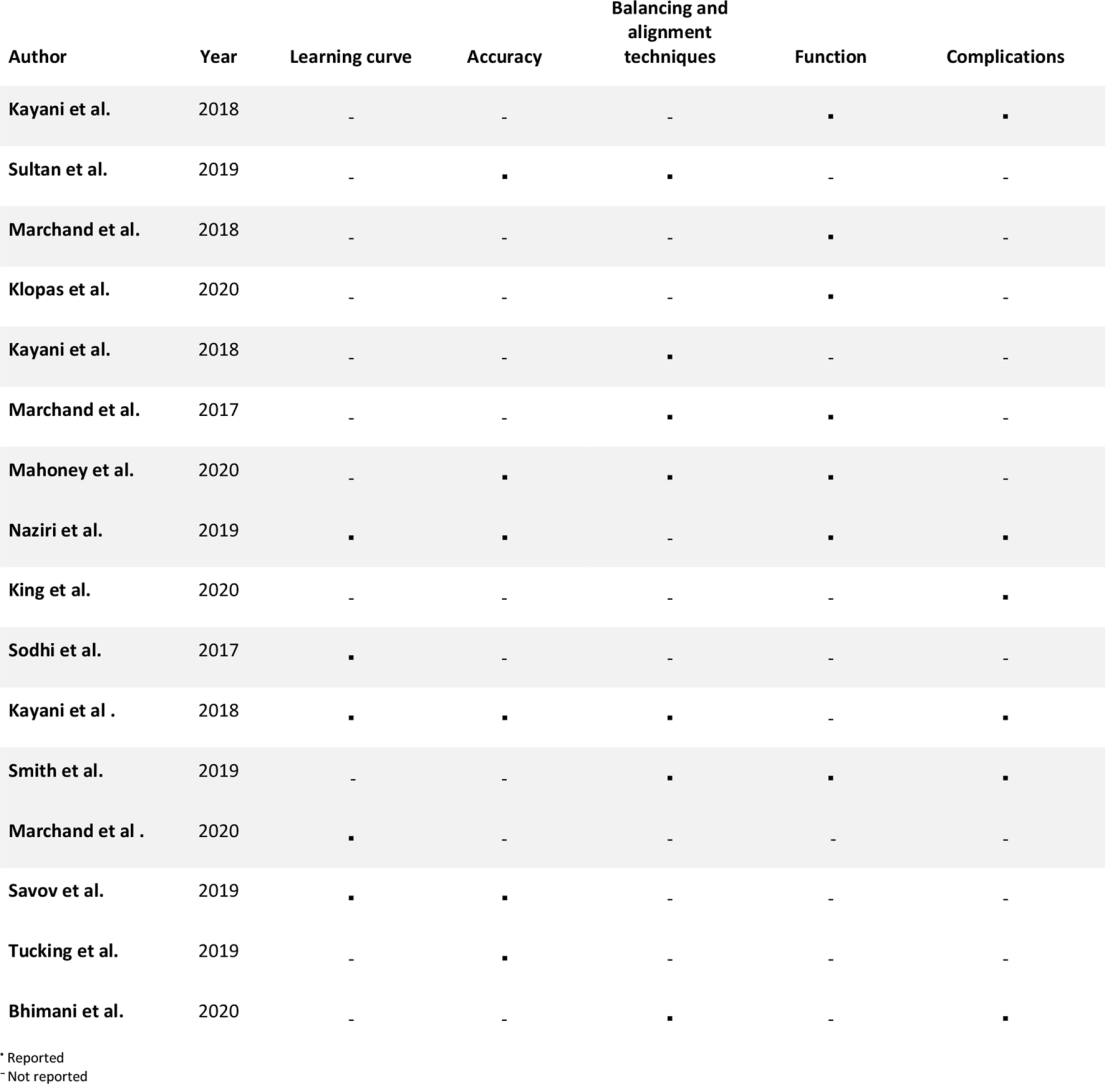
List of studies and the variables reported on

There were 198 articles identified in the initial search of databases and reference lists. After initial screening of titles and abstracts 31 articles met the inclusion criteria for review. On full text screening a further 13 studies were excluded from analysis: five represented articles assessing “fully active” robots [8, 13, 27, 46, 55]; six articles did not have a mTKA control group [21, 33, 37, 41, 48, 56]; four were non-clinical studies (Fig. 1) [12, 24, 35, 42]. A list of the 16 studies which met the inclusion criteria are illustrated in Table 1 [4, 18–20, 23, 25, 28, 30–32, 36, 44, 49–52]. Nine studies were identified from Medline and PubMed, and seven additional studies from Google Scholar (Table 1). The year of publication ranged from 2017 to 2020. Of the 16 published studies identified 11 were prospective and the remainder five were retrospective. There were no randomized controlled trials identified (Table 1, Fig. 2).

**Table 1:** Studies included in the systematic review according to how they were identified, when they were published, design, patient demographics, follow up and type of implant

Authors	Year	Search	Design	Patients (*n* =)	Age	F/U (months)	Implant	Robotic-arm
Bhimani et al. [4]	2020	GS	Retrospective cohorts	140 RATKA vs 127 mTKA	65.4 vs 66.6	1.5	Triathlon PS	Stryker Mako
Kayani et al. [18]	2018	PM/ML	Prospective cohorts	30 RATKA vs 30 mTKA	68.5 vs 69.9	?	Triathlon PS	Stryker Mako
Kayani et al. [19]	2018	PM/ML	Prospective cohorts	40 RATKA vs 40 mTKA	69.7 vs 71.4	1	Triathlon PS (cemented)	Stryker Mako
Kayani et al. [20]	2019	GS	Prospective cohorts	60 RATKA vs 60 mTKA	68.7 vs 67.6	1	Triathlon PS (cemented)	Stryker Mako
Klopas et al. [23]	2020	PM/ML	Prospective cohorts	150 RATKA vs 102 mTKA	68 vs 65	3	Triathlon CR (cemented)	Stryker Mako
King et al. [25]	2020	PM/ML	Retrospective cohort	202 RATKA vs 290 mTKA	68 vs 66	RATKA = 15 vs mTKA = 36	(Reported as same cemented implants only)	Stryker Mako
Mahoney et al. [28]	2020	PM/ML	Prospective cohorts	143 RATKA vs 86 mTKA	64.6 vs 68.5	12	Triathlon CR (cemented)	Stryker Mako
Marchand et al. [30]	2017	PM/ML	Prospective cohorts	28 RATKA vs 20 mTKA	69 vs 67	6	Triathlon CR (cemented)	Stryker Mako
Marchand et al. [31]	2019	PM/ML	Prospective cohorts	53 RATKA vs 53 mTKA	65 vs 63	12	Triathlon CR (cemented)	Stryker Mako
Marchand et al. [32]	2020	GS	Retrospective cohorts	20 RATKA (1 month) vs 60 RATKA (6 months) vs 60 RATKA (1 years) vs 60 mTKA	64 vs 64 vs 65 vs 63	?	Triathlon CR (majority cementless, those done in first month were cemented)	Stryker Mako
Naziri et al. [36]	2019	PM/ML	Retrospective cohorts	40 RATKA vs 40 mTKA	69.5 vs 70.9	3	Triathlon CR (cemented)	Stryker Mako
Savov et al. [44]	2019	GS	Prospective cohorts	30 RATKA vs 30 mTKA	?	?	?	Stryker Mako
Smith et al. [49]	2019	GS	Prospective cohorts	120 RATKA vs 103 mTKA	68 vs 66	12	Triathlon PS (majority cementless, 10 in RATKA and 14 in mTKA cemented due to osteoporosis)	Stryker Mako
Sodhi et al. [50]	2017	GS	Retrospective cohorts	40 RATKA (early) vs 40 RATKA (late) vs 40 mTKA	Not reported	Not reported	Not reported	Stryker Mako
Sultan et al. [51]	2019	PM/ML	Prospective cohorts	43 RATKA vs 39 mTKA	67 vs 66	1 – 1.5	Triathlon CR (cemented)	Stryker Mako
Tucking et al. [52]	2019	GS	Prospective cohorts	40 RATKA vs 40 mTKA	?	?	?	Stryker Mako

*GS* Google Scholar, *ML* Medline, *PM* PubMed, *mTKA* manual Total Knee Arthroplasty, *RATKA* Robotic-arm assisted Total Knee Arthroplasty, ? not stated, *FU* follow up, *N/A* not applicable

### Learning curve (Level of evidence: good)

A total of five studies reported on the learning curve for RATKA (Table 2). All five studies assessed the learning curve for operating time which ranged from 7 to 80 cases [20, 32, 36, 44, 50]. Two studies utilised Cumulative Sum Control Chart (CUSUM) analysis to establish a learning curve inflexion point of proficiency (defined as the point of the learning curve where increasing operating times reverses) which ranged from 7 to 11 cases, dividing the learning curve for RATKA into two distinct phases, Phase 1 (initial learning segment) and Phase 2 (proficiency stage) [20, 44]. Marchand et al. observed continued decreases in mean operative times up to 1 year, after initial adaptation of robotics into workflows, with the mean operative time decreasing by 19 min, and only 12% of the cases exceeded 70 min operating time, quicker than mTKA performed by the same surgeons [32]. Cumulative robotic experience did not impact the accuracy of achieving the planned implant positioning, limb alignment, posterior condylar offset ratio, posterior tibial slope, or native joint line restoration [20]. This was quantified by Kayani et al. and affirmed in other studies which mainly reported no learning curve in accurate post-operative mechanical axis alignment [20, 32].

**Table 2:** Evidence for learning curve

Operating time		
Author	Year	Findings
Kayani et al. [20]	2019	Inflexion point of proficiency after seven cases for operative times (*p* = 0.01) and surgical team anxiety levels (*p* = 0.02)
		Cumulative robotic experience did not affect accuracy of implant positioning (n.s.) limb alignment (n.s.) posterior condylar offset ratio (n.s.) posterior tibial slope (n.s.) and joint line restoration (n.s.)
		Utilising the Surrogate Anxiety Inventory (STAI) questionnaire, Kayani et al. demonstrated that confidence level of surgical team improved in a pattern similar to the learning curve for operative times, with an inflexion point at seven cases. After this point, there was no difference in the overall STAI scores amongst team members between RATKA and mTKA
Marchand et al. [32]	2020	The data indicate a significant decrease in the mean RATKA operative times from 1 month to 1 year of using robotic technology (81 vs. 62 min, *p* < 0.00001)
		The mean surgical times continued to decrease after 6 months of RATKA. In 1 year, the surgeon was performing 88% of the RATKA between 50 and 69 min. The initial cohort and 1-year robotic-assisted mean operative times were 81 and 62 min, respectively (*p* < 0.00001)
		Mean 6-month robotic-assisted operative times were similar to manual times (*p* = 0.12)
Naziri et al. [36]	2019	Proposed point of proficiency as 20 cases
		Intraoperative EBL was comparable between RATKA and traditional TKA cohorts (42.4 vs. 49 ml, *p* = 0.448)
		The RATKA cohort required slightly greater overall surgical time than the traditional TKA cohort (82.5 vs. 78.3 min, *p* = 0.002)
		There was no significant difference in surgical time when comparing the mean surgical time of the second 20 cases of RATKA to the traditional TKA group (81.1 min vs. 78.3 min, *p* = 0.254)
Savov et al. [44]	2019	Inflexion point of proficiency after 11 cases for operative time
		The mean surgery time in the robotic group after finishing the learning curve was 66 min (± 4.2) and in the total manual group 67 min (± 3.5) (n.s.)
Sodhi et al. [50]	2017	Shortening of operative time from 99 min (cases 1–40) to 84 min (cases 81–120)
		No significant differences compared with mTKA in last 40 cases, 84 min vs 81 min

*mTKA* manual total knee arthroplasty, *RATKA* Robotic-arm assisted total knee arthroplasty, *EBL* estimated blood loss

### Component position accuracy (level of evidence: good)

**Fig. 3:**
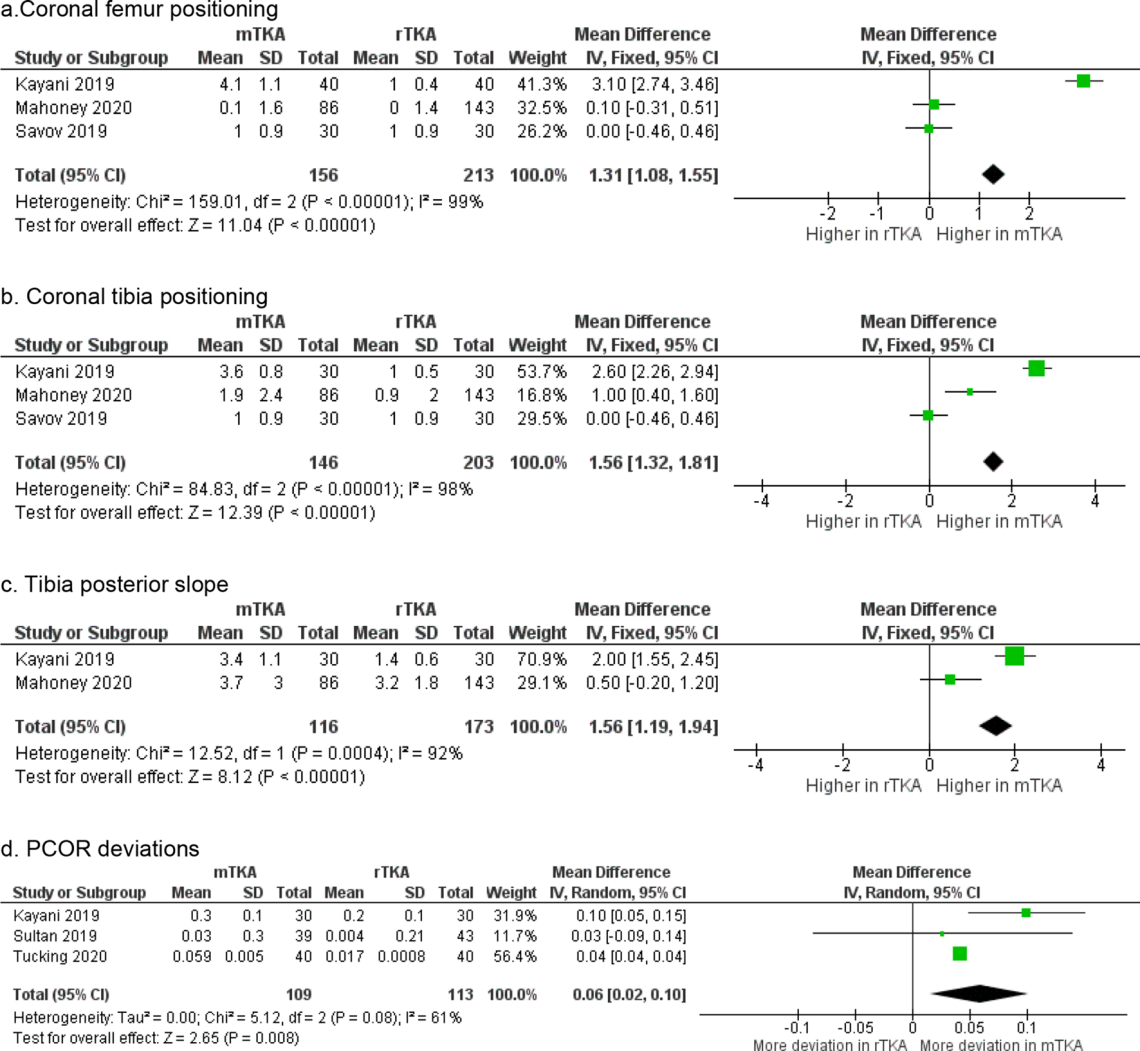
Forest plot of pooled component position accuracy. **a** Coronal femur positioning. **b** Coronal tibia positioning. **c** Tibia posterior slope. **d** PCOR deviations

There were six clinical studies identified that reported on accuracy of component positioning and all compared RATKA against mTKA (Table 3) [4, 20, 44, 50–52]. Three studies reported the posterior condylar offset ratio (PCOR) differences in RATKA and mTKA [20, 51, 52]. There was consistent evidence that RATKA resulted in significantly less differences between pre- and post-operative PCOR. Sultan et al. and Tucking et al. reported the lower differences between pre- and postoperative PCOR (*p* = 0.01 and *p* = 0.001 respectively) [51, 52]. When utilising RATKA, femoral components were placed with increased precision, within mean error range of 0.053 ± 0.020, as compared to 0.072 ± 0.035 when mTKA was utilised (Fig. 3d) [20, 51, 52]. Four studies reported the mechanical and coronal alignment, as well as sagittal alignment accuracies of implant positioning [20, 28, 36, 44]. When utilising RATKA, coronal alignment of the femur was within mean error range of 0.19 ± 1.14 while that for mTKA was 1.3 ± 1.34 (Fig. 3a). For tibia coronal alignment, it was 0.93 ± 1.57 for RATKA and 2.1 ± 1.76 for mTKA (Fig. 3b). As for posterior slope, it was 2.9 ± 1.59 for RATKA and 3.6 ± 2.51 for mTKA (Fig. 3c). These findings showed that RATKA was more precise compared to mTKA. The mTKAs were associated with wider range of component positioning, some of which are outside the preferred mean error range of ± 3 degrees. The pooled results from the forest plots demonstrated that component positioning using RATKA as compared to the mTKA was significantly more accurate (Femur coronal: mean difference 1.31, 95% confidence interval (CI) 1.08–1.55, *p* < 0.00001; Tibia coronal: mean difference 1.56, 95% confidence interval (CI) 1.32–1.81, *p* < 0.00001; Fig. 3a, b). In addition, Mahoney et al. showed that the femoral component external rotation with respect to the transepicondylar axis was more precise with the use of RATKA although this was not statistically significant (*p* = 0.195) [28].

**Table 3:** Evidence for implant accuracy

Component accuracy		
Kayani et al. [20]	2019	*[Accuracy in achieving the planned implant positions compared to conventional jig based TKA]*
		Mechanical alignment (degrees), mean (SD)
		mTKA 3.2 ± 1.2 vs RATKA 1.5 ± 0.9, *p* < 0.001
		PCOR
		mTKA 0.3 ± 0.1 vs RATKA 0.2 ± 0.1, n.s
		Posterior slope
		mTKA 3.4 ± 1.1 vs RATKA 1.4 ± 0.6, *p* < 0.001
		Joint line
		mTKA 2.9 ± 1.4 vs RATKA 1.0 ± 0.6, *p* < 0.001
		Femur coronal
		mTKA 4.1 ± 1.1 vs RATKA 1.0 ± 0.4, *p* < 0.001
		Femur sagittal
		mTKA 4.2 ± 0.8 vs RATKA 2.1 ± 0.7, *p* < 0.001
		Tibia coronal
		mTKA 3.6 ± 0.8 vs RATKA 1.0 ± 0.5, *p* < 0.001
		Tibia sagittal
		mTKA 3.9 ± 1.0 vs RATKA 2.0 ± 0.6, *p* < 0.001
Mahoney et al. [28]	2020	Coronal positions measured via CT (mean ± SD)
		Femoral components
		mTKA 0.1 (± 1.6) varus vs RATKA 0.0 (± 1.4) varus, *p* = 0.506
		Tibial components
		mTKA 1.9 (± 2.4) varus vs RATKA 0.9 (± 2.0) varus, *p* = 0.005
		Femoral component external rotation relative to the transepicondylar axis
		mTKA 1.1 ± 2.3 vs RATKA 0.5 ± 2.3 degrees, (*p* = 0.195)
		Tibial slopes
		mTKA 3.7 ± 3.0 vs 3.2 ± 1.8 degrees, (*p* = 0.291)
Naziri et al. [36]	2019	Postoperative alignment was within + 3.0° of the mechanical axis for all patients in both RATKA and mTKA groups
Savov et al. [44]	2019	Limb alignment and restoration of the joint line
		mTKA no difference from RATKA
		RATKA deviation limb alignment to the intraoperative plan, mean = 2° (± 1.1)
		RATKA deviation of the medial proximal tibial (mPTA) and distal lateral femoral angle (dLFA) = 1° (± 0.9) for both
Sultan et al. [51]	2019	Postoperative PCOR, mean (SD)
		mTKA 0.53 (± 0.3) vs RATKA 0.49 (± 0.21), *p* = 0.024
		Difference between pre- and postoperative PCOR, mean
		mTKA 0.03 vs RATKA 0.004, *p* = 0.01
		Postoperative ISI outside of the normal range (0.8–0.12)
		mTKA 12 vs RATKA 4
Tucking et al. [52]	2020	Postoperative PCOR, mean (SD)
		mTKA 0.47 (± 0.05) vs RATKA 0.51 (± 0.05), *p* = 0.006)
		PCOR (difference between pre- and postoperative), mean
		mTKA 0.059 vs RATKA -0.017, *p* = 0.001
		Relative deviation of PCOR, mean (SD)
		mTKA 12.03% (± 9.1) vs RATKA 3.9% (± 4.5)

*PCOR* Posterior condylar offset ratio, *ISI* Insall-Salvati Index, *mTKA* manual total knee arthroplasty, *RATKA* Robotic-arm assisted total knee arthroplasty

### Knee balancing and alignment techniques (level of evidence: fair)

Seven (44%) of the 16 clinical studies reported the balancing and/or the alignment techniques utilised for the TKAs groups (Table 4). Out of the seven studies, two stated that the mTKAs were performed using a standard measured resection technique followed by soft tissue releases to achieve a balanced and mechanically aligned knee, but did not define how the RATKA groups were balanced or how the alignment was achieved and whether it was using mechanical, kinematic or restricted kinematic alignment methods [30, 51]. Two studies used same methods in both groups, Bhimani et al. using gap balancing techniques and Mahoney et al. using measured resection techniques [4, 28]. The three remaining studies, however, used different methods for their RATKA and mTKA groups, namely kinematic alignment and gap balancing methods for RATKAs and measured resection methods for mTKAs [18, 20, 49].

**Table 4:** Evidence for alignment and balancing techniques

Author	Year	Findings
Bhimani et al. [4]	2020	RATKA: to achieve the desired bone cuts and target limb alignment, along with symmetrically balanced flexion and extension gaps
		Unknown which technique and which reference alignment was utilised
		mTKA: A gap balancing technique was utilized using a ligamentous tensioning device with the extension gap balanced followed by balancing the flexion gap after release of the posterior cruciate ligament
		Gap balancing techniques utilised
Kayani et al. [18]	2018	mTKA utilised a measured resection technique aligned to the mechanical axis
		RATKA utilised dynamic referencing to achieve equal gaps throughout the range of motion, utilising gap balancing and kinematic alignment techniques
Kayani et al. [19]	2018	RATKA: intraoperative dynamic gap balancing techniques were used with kinematic alignment assessed through the arc of motion, and enabled fine tuning of implant positioning based on laxity of the soft tissue envelope, within 2 mm of the planned bone resection
		Utilised restricted kinematic alignment techniques
		mTKA: measured resection and mechanical alignment as reference
Mahoney et al. [28]	2020	Both RATKA and mTKA utilised mechanical alignment as reference for all except nine cases of two centers that were targeted within ± 3 degrees
Marchand et al. [30]	2017	RATKA: the prosthesis was manipulated allowing for optimal balancing and realignment. The knee was brought into extension, and alignment was checked with the robotic-assisted device both in extension and at 90 degrees of flexion
		No mention if mechanical/kinematic alignment was utilised to check the knee in extension
		mTKA: measured resection techniques used with mechanical alignment as reference
Smith et al. [49]	2019	RATKA: equal gap measurements within 1 mm between the flexion and extension gaps and the medial and lateral gaps, keeping limb alignment within 3 degrees of the mechanical axis and use the bone cuts to balance gaps instead of soft tissue releases unless the target fell out of 3 degrees window, at which point a combination of bone cuts and soft tissue releases was utilized to achieve balanced gaps within 1 mm
		Restricted kinematic alignment and gap balancing techniques
		mTKA: Mechanical alignment and measured resection techniques utilised
Sultan et al. [51]	2019	RATKA: Intraoperative adjustments to the plan were performed to determine ideal component placement for a balanced knee. Ligament balancing was assessed following resections and after trialing
		mTKA was performed using a standard technique
		No mention of measured resection or gap balancing techniques nor how mechanical / kinematic alignment was achieved

*mTKA* manual total knee arthroplasty, *RATKA* Robotic-arm assisted total knee arthroplasty

### Functional outcomes (level of evidence: good)

**Fig. 4:**
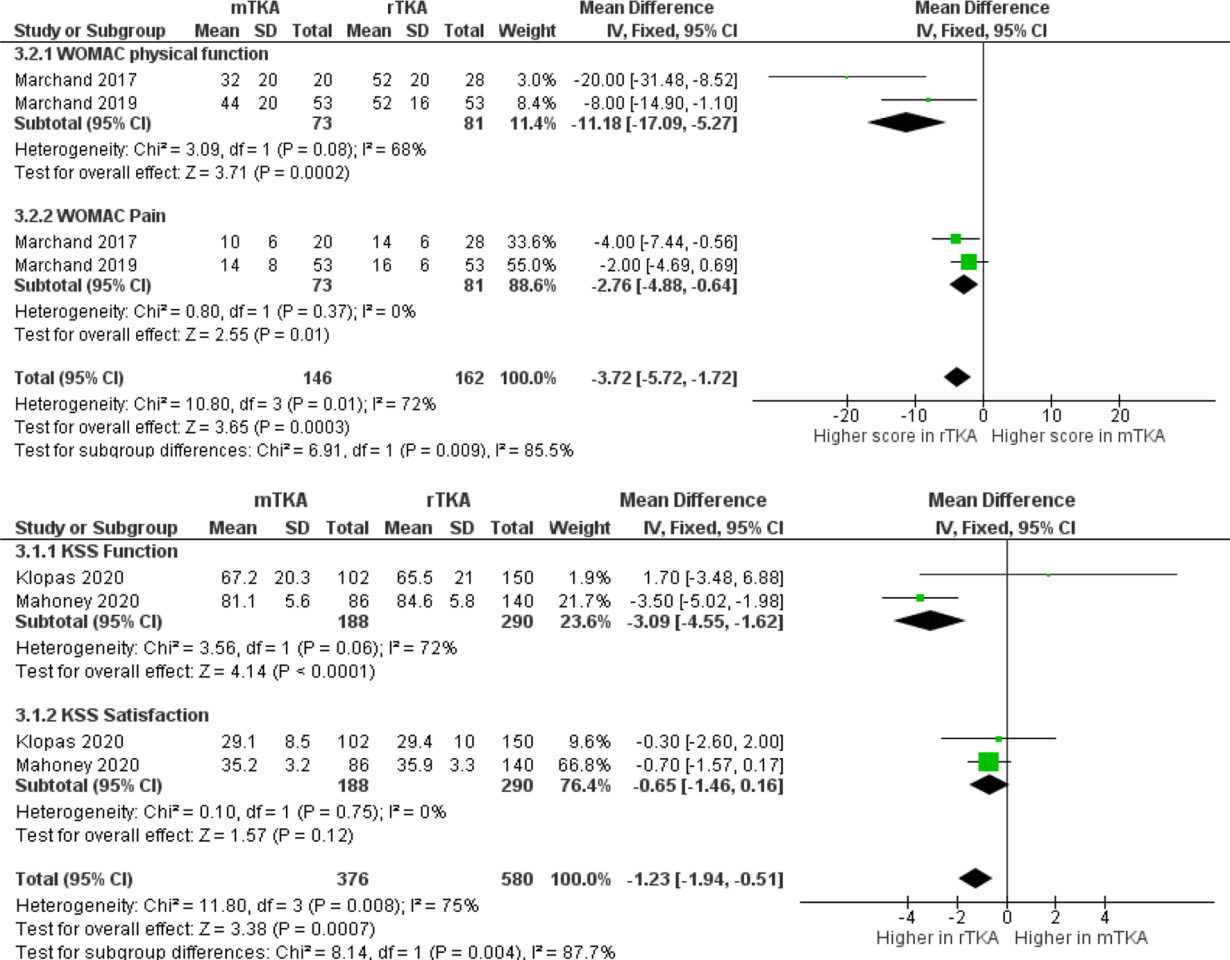
Forest plot of pooled functional outcome scores

There were seven clinical studies reporting the functional outcomes following RATKA compared to mTKA (Table 5) [19, 23, 28, 30, 31, 35, 49]. Different outcome scores were utilised across the included studies, with the Knee Society Scores (KSS) being the most reported followed by Western Ontario and McMaster Universities Osteoarthritis Index (WOMAC) scores. Overall KSS ranged from 44.5 to 86.5-points and WOMAC scores ranged from 40 to 92-points in the RATKA group, whereas in mTKA group, it was 46.9 to 87.5-points for KSS scores and 16 to 86-points in WOMAC scores (Fig. 4). Khlopas et al. found evidence to suggest significant differences in the improvements made in scores but not in the absolute mean postoperative KSS scores achieved, while Mahoney et al. observed better scores in RATKA but it was not statistically significant (*p* = 0.159) [23, 28]. Meta-analysis of outcome data from the studies demonstrated RATKA resulted in a significantly better KSS score compared to mTKA in the short- to mid-term follow-up (mean difference 1.23, 95% CI 0.51–1.94, *p* = 0.004: Fig. 4). Meta-analysis of outcome data for WOMAC scores also demonstrated RATKA had significantly better scores compared to mTKA in the short- to mid-term follow-up (mean difference 3.72, 95% CI 1.72–5.72, *p* = 0.009: Fig. 4).

**Table 5:** Evidence for functional outcomes

Author	Year	Findings
Kayani et al. [19]	2018	Shorter time to straight leg raise
		mTKA 31.0 (IQR 24.0–44.0) vs RATKA 20.0 (IQR 18.0–21.0), *p* < 0.001
		Improved maximum knee flexion at discharge
		mTKA 93.3 (90.0–110.0) vs RATKA 104.1 (90.0–120.0), *p* < 0.001
		Mean pain score – Day 3, mean (range)
		mTKA 4.5 (2.0–7.0) vs RATKA 2.6 (1.0–5.0)
Khlopas et al. [23]	2020	KSS scores (Improvements in functional activity walking and standing), 4 to 6 weeks postoperatively, mean
		mTKA 1.2 vs RATKA 1.4 (*p* = 0.019)
		KSS (functional activity score), 3 months, mean (estimated SD)
		mTKA 67.2 (± 21) vs RATKA 65.5 (± 20.3), *p* = 0.613
		KSS Pain scores improvements, 3 months, mean
		Walking
		mTKA 4.1 vs RATKA 4.3, *p* = 0.06
		Total symptoms
		mTKA 10.3 vs RATKA 10.5, *p* = 0.781
		KSS Patient Satisfaction Scores Mean (estimated SD)
		4–6 weeks:
		mTKA 25.9 (± 8) vs RATKA 25.2 (± 10), *p* = 0.268
		3 months:
		mTKA 29.1 (± 8.5) vs RATKA 29.4 (± 10), *p* = 0.268
Mahoney et al. [28]	2020	*[For all PROMs, longitudinal trends mTKA and RATKA similar at 1 year except VR12 physical scores]*
		KSS Adjusted mean (95% C.I.)
		symptoms
		mTKA 20.3 [18.4, 22.2] vs RATKA 20.8 [18.9, 22.7] *p* = 0.531
		Satisfaction
		mTKA 35.2 [32.0, 38.4] vs RATKA 35.9 [32.6, 39.2] *p* = 0.532
		Expectations
		mTKA 10.6 [9.2, 12.0] vs RATKA 11.2 [9.7, 12.7] *p* = 0.192
		Function
		mTKA 81.1 [75.5, 86.8] vs RATKA 84.6 [78.8, 90.4] *p* = 0.159
		Veterans RAND 12-item health scale Adjusted mean (95% C.I.)
		physical component
		mTKA 50.5 [47.5, 53.5] vs RATKA 52.9 [49.9, 55.9] *p* = 0.034
		Mental component
		mTKA 56.0 [53.7, 58.3] vs RATKA 54.6 [52.2, 57.0] *p* = 0.213
Marchand et al. [30]	2017	WOMAC (physical function), 6-month postoperative, mean (SD)
		mTKA 9 (± 5) vs RATKA (4 ± 5), *p* = 0.055
		WOMAC (pain), 6-month postoperative, mean (SD)
		mTKA 5 (± 3) vs RATKA 3 (± 3) *p* < 0.05
		Converted WOMAC scores
		mTKA 32(20) vs RATKA 52(20)
		mTKA 10(6) vs RATKA 14(6)
		Total: mTKA 42 vs RATKA 76
Marchand et al. [31]	2019	WOMAC (Physical Function), mean (SD)
		mTKA 6 (± 5) points vs RATKA 4 (± 4) points, *p* < 0.05
		WOMAC (Pain scores), mean (SD)
		mTKA 3 (± 4) points, vs RATKA 2 (± 3) points, *p* = 0.06
		Converted WOMAC scores
		PF: mTKA 44 (20) vs RATKA 52(16)
		PS: mTKA 14(8) vs RATKA 16(6)
		Total: mTKA 58 vs RATKA 78
Naziri et al. [36]	2019	Range of motion improvement at 90 days
		mTKA -8.7 deg vs RATKA + 3.8 deg, *p* < 0.05
		KSS at 30, 60, 90 days, mean
		mTKA 90.9 vs RATKA 86.0, *p* = 0.082
		mTKA 91.7 vs RATKA 91.9 *p* = 0.938
		mTKA 89.5 vs RATKA 88.2 *p* = 0.730
		LEAS at 30, 60, 90 days
		mTKA 11.50 vs RATKA 11.63, *p* = 0.736
		mTKA 11.65 vs RATKA 12.06, *p* = 0.271
		mTKA 11.94 vs RATKA 12.18, *p* = 0.519
		*[No report on the spread of data hence not included in the meta-analysis]*
Smith et al. [49]	2019	Range of motion
		mTKA 1 to 116 degrees vs RATKA 0 to 119 degrees (*p* = 0.88 and *p* = 0.02 for extension and flexion)
		KSS function score (6-week and 1-year average score)
		mTKA 58 and 73 vs RATKA 63 and 80, *p* = 0.02 and *p* = 0.005)
		1-year KSS knee score
		mTKA 82 vs RATKA 85, *p* = 0.046
		Overall patient reported satisfaction reported with a Likert scoring system
		mTKA 82% vs RATKA 94% (*p* = 0.036)
		KSS Patient satisfaction scores mean
		mTKA 6.6 vs RATKA 7.1, (*p* = 0.027)
		*[No report on the spread of data hence not included in the meta-analysis]*

WOMAC: Western-Ontario and McMaster Universities Arthritis Index, KSS: Knee society score, LEAS: Lower extremity activity scale, mTKA: manual total knee arthroplasty, RATKA: Robotic-arm assisted total knee arthroplasty

### Complications (level of evidence: good)

**Fig. 5:**
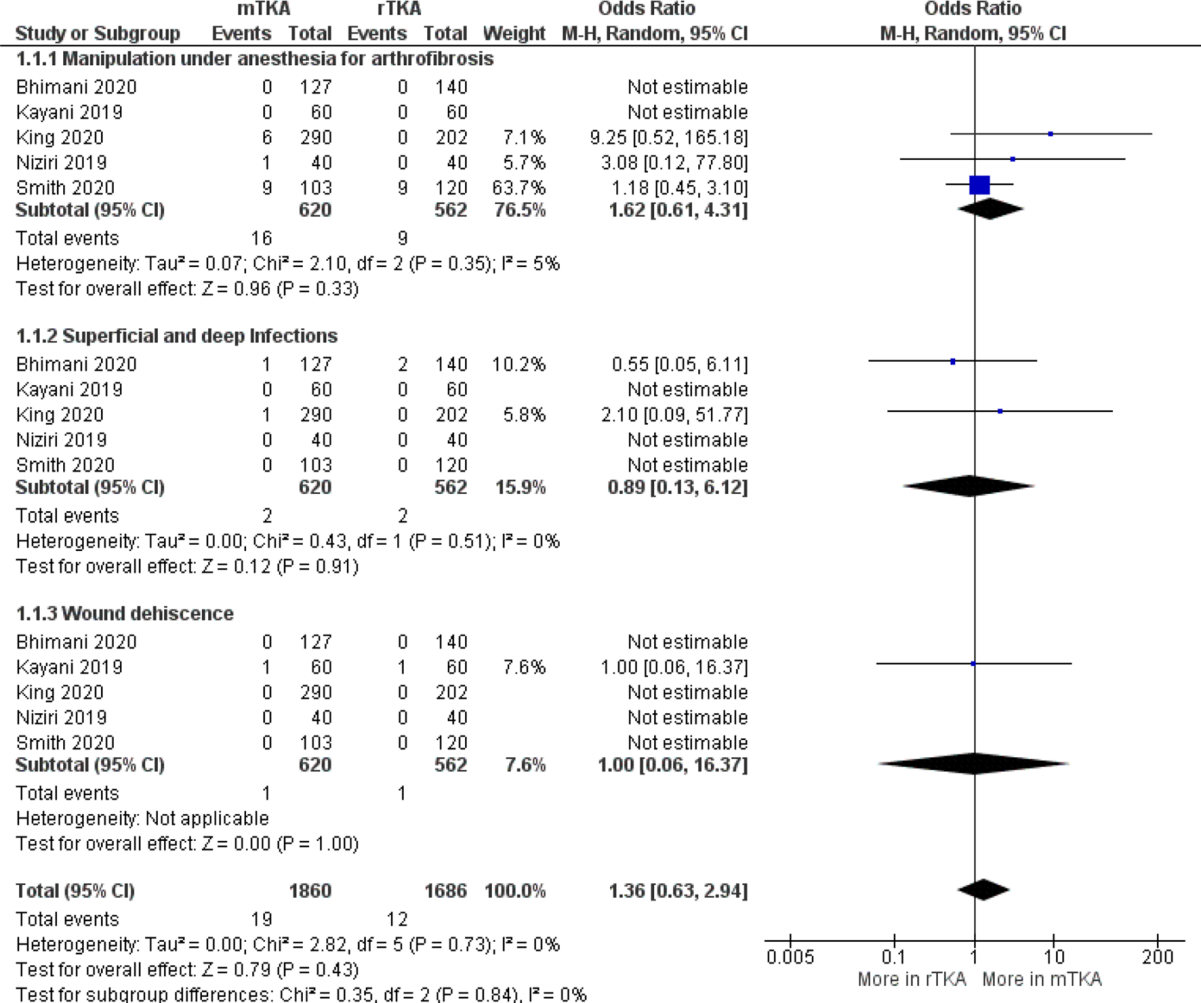
Forest plot of pooled complications

Six studies reported on the complications between RATKA and mTKA groups (Table 6) [4, 19, 20, 25, 36, 49]. The most reported complications were arthrofibrosis requiring MUA, superficial or deep infections and wound dehiscence. Overall complication rates were low. No studies reported any pin site fractures or component revisions. In the RATKA group, arthrofibrosis rates ranged from 0 to 7.5%, superficial or deep infection rates ranged from 0 to 1.4% and wound dehiscence rates ranged from 0 to 2.5%. In mTKA groups arthrofibrosis rates ranged from 0 to 8.7%, superficial or deep infection rates ranged from 0 to 0.8%, and wound dehiscence rates ranged from 0 to 2.5%. A forest plot of pooled reported complication data demonstrated that there was no difference in arthrofibrosis, infection or wound dehiscence rates, but there was a higher risk (odds ratio 1.36, 95% confidence interval (CI) 0.63 to 2.94, *p* = 0.84; Fig. 5) for overall complication rate associated with mTKA compared to RATKA in short-term follow-up, but this was not significant.

**Table 6:** Evidence for complications

Author	Year	Findings
Bhimani et al. [4]	2020	Pain scores (mean) – 2 weeks
		At rest
		mTKA 3.5 vs RATKA 2.6, *p* = 0.001
		With activity
		mTKA 7.0 vs RATKA 6.3, *p* = 0.03
		Pain scores (mean) – 6 weeks
		At rest
		mTKA 1.6 vs RATKA 1.0, *p* = 0.03
		With activity
		mTKA 4.7 vs RATKA 3.8, *p* = 0.02
		Time to discharge (days)
		mTKA 2.3 vs RATKA 1.9, *p* < 0.001
		MUA
		mTKA 0 vs RATKA 0
		Superficial and deep infections
		mTKA 1 and RATKA 2 (prosthetic joint infections)
		Wound dehiscence
		mTKA 0 vs RATKA 0
Kayani et al. [19]	2018	MUA
		mTKA 0 vs RATKA 0
		Superficial and deep infections
		mTKA 0 vs RATKA 0
		Wound dehiscence
		mTKA, 1 (distal part of the midline incision) vs RATKA group 1 (incision for the proximal tibial registration pins), all recovered with regular dressings and prophylactic oral antibiotics
		Time to discharge (hrs), median (IQR)
		mTKA 105.0 (IQR 98.0–126.0) vs RATKA 77.0 (IQR 74.0–81.0), p < 0.001
		Post-operative pain score – day 3, mean (range)
		mTKA 4.5 (2.0–7.0) vs RATKA 2.6 (1.0–5.0), *p* < 0.001
Kayani et al. [20]	2019	MUA
		mTKA 0 vs RATKA 0
		Superficial and deep infections
		mTKA 0 vs RATKA 0
		Wound dehiscence
		mTKA, 1 (distal part of the midline incision) vs RATKA group 1 (incision for the proximal tibial registration pins), all recovered with regular dressings and prophylactic oral antibiotics
King et al. [25]	2020	Pain scores, mean
		mTKA 5.1 vs RATKA 5.5, *p* = 0.288
		Time to discharge, days
		mTKA 2.6 vs RATKA 2.3, *p* < 0.001
		Early readmissions
		mTKA 4 vs RATKA 3, *p* = 0.9
		Most common reason for readmission across both groups was related to bleeding events (3/7)
		Return to ED
		mTKA 21 vs RATKA 10, *p* = 0.30
		Most common reason was leg swelling, this accounted for only 1/10 (10%) of RATKA while it accounted for 7/21 (33%) of mTKA (*p* = 0.17)
		MUA
		mTKA 6 vs RATKA 0
		Superficial and deep infections
		mTKA 1 vs RATKA 0
		Wound dehiscence
		mTKA 0 vs RATKA 0
Naziri et al. [36]	2019	Time to discharge (days), median (IQR not reported)
		mTKA 1.92 vs RATKA 1.27, *p* < 0.001
		30 days
		Minor Complications—None reported
		Major Complication Rate
		mTKA 2.5% vs RATKA 0.0%, *p* = 0.320
		*[mTKA with Arthrofibrosis requiring Manipulation under Anesthesia (MUA) (n* = *1)]*
		MUA
		mTKA 1 vs RATKA 0
		Superficial and deep infections
		mTKA 0 vs RATKA 0
		Wound dehiscence
		mTKA 0 vs RATKA 0
		*No complications for 60 and 90 day follow ups*
Smith et al. [49]	2019	Time to discharge (days)
		mTKA 3 vs RATKA 2
		MUA
		mTKA 9 vs RATKA 9
		*[If patients did not reach active range of motion to 105 degrees of flexion at 6 weeks then manipulation was recommended.]*
		Superficial and deep infections
		mTKA 0 vs RATKA 0
		Wound dehiscence
		mTKA 0 vs RATKA 0
		Non-fatal pulmonary embolism
		mTKA 0 vs RATKA 2

*KSS* Knee Society Score, *IQR* Inter-quartile range, *ED* Emergency Department, *MUA* Manipulations Under Anaesthesia, *mTKA* manual Total Knee Arthroplasty, *RATKA* Robotic-arm assisted Total Knee Arthroplasty

## Discussion

The key findings of this review were the following: (a) the learning curve of RATKA for surgical proficiency, stress and confidence levels was short (7–11 cases); (b) component positioning for RATKA was more precise when compared with mTKA; (c) short-term patient-reported outcomes were better with RATKA; (d) RATKA had similar complication rates as mTKA in the short-term and (e) there is a need for improved reporting of knee alignment and balancing techniques when comparing RATKA and mTKA.

The barrier for initial adoption is considered low for RATKA as the learning curve for RATKA was short. Decreasing operating times were noted after the first 7 to 11 cases, following initial phases of adaptation in techniques [20, 44]. This is coupled with the fact that there is no learning curve for accuracy of implant positioning. However, to achieve higher proficiencies, a relatively wider range in overall number of cases, 20 to 80, was required. The use of a defined methodology such as the CUSUM analysis is preferable as it identifies one exact turning point over a continuous curve, shown to be an accepted method in exploring the actual learning curve [20, 22, 54]. However, to gain mastery of RATKA techniques, with surgeons becoming more comfortable and experienced, it would be after about 80 cases, with reported consistently shorter operating times thereafter [32].

RATKAs have a theoretical advantage over mTKAs in component positioning in all three dimensions: coronal, sagittal and axial. The current analysis showed consistent evidence that RATKA resulted in less outliers in the component positions. This reflects the precision of the technique, irrespective of the knee alignment and balancing techniques used, which may differ from surgeon to surgeon. With the higher accuracy of component placement by RATKA, especially in the sagittal plane, the surgeon could potentially gap balance the knee more precisely with the use of RATKA compared to mTKA. Traditionally, balanced gaps have been considered a prerequisite for good function and endurance in TKA, and a balanced knee could affect long-term clinical outcomes [34, 47]. Furthermore, this precision and intra-operative feedback on the gap balancing could possibly minimise limitations in the conventional techniques and sizing options. Kayani et al. reported better early maximum knee flexion for RATKA 104.1 (90.0–120.0) degrees compared to mTKA 93.3 (90.0–110.0) degrees (*p* < 0.001), and there was a trend towards a reduced incidence of stiffness post-operatively (requiring MUA) for the RATKA cohort [19]. Future longer-term studies reporting the clinical outcomes of RATKAs should include such measures and evaluate the clinical relevance.

There is a need for improved reporting criteria for alignment and balancing techniques used in studies comparing RATKA with mTKA. Of the seven clinical studies that reported balancing and alignment techniques, only two studies used same balancing and alignment methods and two studies did not even state how their RATKAs groups were balanced or aligned [4, 28, 30, 51]. RATKA appear to be highly effective in improving the precision of restricted kinematic alignment techniques. Kayani et al. utilised measured resection for mTKA and restricted kinematic alignment for RATKA and recorded a significantly better component position accuracy in all three variables, namely coronal femur and tibia position and tibia posterior slope [20]. Mahoney et al. on the other hand utilised measured resection technique in both RATKA and mTKA, and better accuracies was shown only in tibia coronal positioning. However, due to inconsistent reporting methods in the stated technique for RATKA, there are currently insufficient data to indicate whether the restricted kinematic alignment technique yields better results for RATKAs. Overall, there is a need for improved reporting of the alignment and balancing techniques used (a critical aspect of comparing manual and robotic techniques). Future studies should report this to allow better conclusions to be made on the best alignment and balancing technique to be used with RATKA.

The current meta-analysis has demonstrated improved short-term patient-reported outcome measures (PROMs) for RATKA, compared to mTKA, according to the pooled KSS and WOMAC scores. Although there was a significant difference in the KSS of 1.23 points in favour of RATKA, this is not greater than the minimal clinical important difference (MCID) and, therefore, may not be clinically relevant [26]. Similarly, for WOMAC scores, there was a statistically significant difference but may not be clinically relevant [10]. Mahoney et al. reported on better physical scores of VR-12 in favour of RATKA, and again this was also not greater than the MCID [28, 38]. Although this may suggest that RATKA techniques may not make clinical differences in the short and medium term, it may also be reflective of the intrinsic limitations of the PROMs used, especially the ceiling effect. Scores such as the Forgotten joint score have a limited ceiling effect and may be better at demonstrating measurable clinically significant differences between RATKA and mTKA in future studies [11, 14].

Overall, the number of complications is low. Despite pooling together 1674 cases, only 24 arthrofibrosis requiring MUA, four infections, two wound dehiscence and no pin-site fractures were identified. The pooled results showed that the improved accuracy of component position obtained with RATKAs was not associated with any reduced joint stiffness nor was there increased risks of wound dehiscence or peri-prosthetic fractures. Given that only 33% of the weighted studies (*n* = 2/6) in the current analysis had a minimum follow-up of 1 year, of which only one had a follow-up of 15 months for the RATKA group, there remains a need for improved evidence with longer follow-up to better assess longer term complication and revision rates.

There are a few key limitations of the data set. First, the inclusion criteria, such as English language, may have excluded relevant studies. Second, the methodology has known limitations regarding the type of studies included (non-blinded, non-randomised prospective and retrospective cohort studies) and the difficulties in assessing the analyses without access to the raw data. Third, there was an important variability between the studies with respect to the type of outcome measurement used, the follow-up period and cohorts evaluated. Moreover, there are not yet any published randomized controlled trials. The studies on RATKAs are few and mainly have short-term follow-up. Future studies with longer term follow-up will be needed to provide more conclusive findings in assessing the outcomes and benefits. Another limitation was that two studies were excluded in the forest plot for functional outcomes of KSS scores because the spread of the data was not available for pooled analysis [36, 49]. Furthermore, the overall WOMAC score collected by Marchand et al. used a modified scale rather than the original WOMAC [30, 31]. These may have introduced bias into the analysis.

The main strength of this study, compared to previous systematic reviews, was the quantitative assessment of a singular image-based RATKA system. The assessments made with regard to the accuracy of component positioning in the various parameters allow the surgeon to be fully aware of the strengths and weaknesses of the system, modifying techniques to fine-tune bone resection, implant positioning and soft tissue releases to achieve the desired alignment and balancing, which was shown to be associated with improved functional outcomes.

## Conclusion

RATKA demonstrated improved accuracy of component positioning and early patient-reported outcomes, though it may not be clinically significant. The learning curve of RATKA for operating time was between 7 and 11 cases. Future well-powered studies should report on the knee alignment and balancing techniques utilised in RATKAs to enable greater comparisons to be made on which techniques maximally benefit patient outcomes and provide better insights into alternate alignment philosophies.

## Abbreviations

RATKA: Robotic-arm assisted total knee arthroplasty
mTKA: Manual total knee arthroplasty
CUSUM: Cumulative sum control chart
CI: Confidence interval
TKA: Total knee arthroplasty
PRISMA: Preferred reporting items for systematic review and meta-analysis
NIH: National institutes of health
KSS: Knee society scores
WOMAC: Western Ontario and McMaster University Osteoarthritis Index
MUA: Manipulation under anaesthesia
STAI: Surrogate anxiety inventory
PCOR: Posterior condylar offset ratio
PROMs: Patient reported outcome measures
MCID: Minimal clinical important difference
