# Correction to: Robotic‑arm assisted total knee arthroplasty is associated with improved accuracy and patient reported outcomes: a systematic review and meta‑analysis

**DOI:** 10.1007/s00167-021-06522-x

**Published:** 2021-04-18

**Authors:** Junren Zhang, Wofhatwa Solomon Ndou, Nathan Ng, Paul Gaston, Philip M. Simpson, Gavin J. Macpherson, James T. Patton, Nicholas D. Clement

**Affiliations:** Department of Orthopedics and Trauma, The Royal Infirmary of Edinburgh, Little France, Edinburgh, EH16 4SA UK

## Correction to: Knee Surgery, Sports Traumatology, Arthroscopy 10.1007/s00167-021-06464-4

Authors would like to correct the error in abstract. Correct version of abstract updated here.

The original article has been corrected.

**Abstract**

This systematic review and meta-analysis were conducted to compare the accuracy of component positioning, alignment and balancing techniques employed, patient-reported outcomes, and complications of robotic-arm assisted total knee arthroplasty (RATKA) with manual TKA (mTKA) and the associated learning curve. Searches of PubMed, Medline and Google Scholar were performed in October 2020 using PRISMA guidelines. Search terms included “robotic”, “knee” and “arthroplasty”. The criteria for inclusion were published clinical research articles reporting the learning curve for RATKA and those comparing the component position accuracy, alignment and balancing techniques, functional outcomes, or complications with mTKA. There were 198 articles identified, following full text screening, 16 studies satisfied the inclusion criteria and reported the learning curve of rTKA (*n* = 5), component positioning accuracy (*n* = 6), alignment and balancing techniques (*n* = 7), functional outcomes (*n* = 7), or complications (*n* = 5). Two studies reported the learning curve using CUSUM analysis to establish an inflexion point for proficiency which ranged from 7 to 11 cases and there was no learning curve for component positioning accuracy. The meta-analysis showed a significantly lower difference between planned component position and implanted component position, and the spread was narrower for RATKA compared with the mTKA group (Femur coronal: mean 1.31, 95% confidence interval (CI) 1.08–1.55, *p* < 0.00001; Tibia coronal: mean 1.56, 95% CI 1.32–1.81, *p* < 0.00001). Three studies reported using different alignment and balancing techniques between mTKA and RATKA, two studies used the same for both group and two studies did not state the methods used in their RATKA groups. RATKA resulted in better Knee Society Score compared to mTKA in the short-to-mid-term follow up (95%CI [− 1.23, − 0.51], *p* = 0.004). There was no difference in arthrofibrosis, superficial and deep infection, wound dehiscence, or overall complication rates. RATKA demonstrated improved accuracy of component positioning and patient-reported outcomes. The learning curve of RATKA for operating time was between 7 and 11 cases. Future well-powered studies on RATKAs should report on the knee alignment and balancing techniques utilised to enable better comparisons on which techniques maximise patient outcomes.

*Level of evidence* III.

